# Reflecting on the methodological challenges of recruiting to a United Kingdom-wide, multi-centre, randomised controlled trial in gynaecology outpatient settings

**DOI:** 10.1186/1745-6215-14-389

**Published:** 2013-11-15

**Authors:** Sylvia Dickson, Janet Logan, Suzanne Hagen, Diane Stark, Cathryn Glazener, Alison M McDonald, Gladys McPherson

**Affiliations:** 1 Nursing, Midwifery and Allied Health Professions Research Unit, Glasgow Caledonian University, Buchanan House, Cowcaddens Road, Glasgow G4 0BA, UK; 2 Functional Bowel Service, Colorectal/Stoma Care Office, Clinic 2, Balmoral Building, Leicester Royal Infirmary, Infirmary Square, Leicester LE1 3WW, UK; 3 Health Services Research Unit, University of Aberdeen, Health Sciences Building, Foresterhill, Aberdeen AB25 2ZD, UK

**Keywords:** Randomised controlled trial, Recruitment, Barriers, Gynaecology

## Abstract

**Background:**

Successful recruitment of participants to any trial is central to its success. Trial results are routinely published, and recruitment is often cited to be slower and more difficult than anticipated. This article reflects on the methodological challenges of recruiting women with prolapse attending United Kingdom (UK) gynaecology outpatient clinics to a multi-centre randomised controlled trial (RCT) of physiotherapy, and the systems put in place in an attempt to address them.

**Methods:**

Gynaecology outpatients with symptomatic prolapse were to be recruited over a 16-month period from 14 UK hospitals and one New Zealand hospital. Eligible women were informed about the trial by their gynaecologist and informed consent was obtained by the central trial office. Recruitment difficulties were encountered early on, and a number of strategies were employed to try to improve recruitment.

**Results:**

Some strategies were more successful than others and they differed in the resources required. Actions that facilitated recruitment included increasing recruiting centres to 23 UK and two international hospitals, good centre support, using processes embedded in clinical practice, and good communication between the trial office, collaborators and participants. Collaborator incentives, whereby staff involved received the benefit immediately, were more successful than a nominal monetary payment per woman randomised. Barriers to recruitment included fewer eligible women than anticipated, patient’s preference to receive active treatment rather than allocation to the control group, lack of support staff and high staff turnover. Geographical variations in Primary Care Trust Research Management and Governance approval systems and general practitioner (GP) referral procedures also impacted negatively on recruitment.

**Conclusions:**

Our article reflects on the methodological challenges of recruiting to a multi-centre RCT in a UK gynaecology setting. Effective interventions included increasing the number of recruiting centres and providing collaborator incentives. Barriers to recruitment included fewer eligible women than anticipated, patient’s preference to be allocated to the treatment group, lack of support staff, and variations in approval systems and GP referral procedures. To improve the evidence base on clinical trial recruitment, trialists need to publish their experiences and lessons learned. Future RCTs should evaluate, where possible, the effect of strategies designed to improve recruitment and retention.

**Trial registration:**

Current Controlled Trials ISRCTN35911035

## Background

Randomised Controlled Trials (RCTs) are considered to be the gold standard for evaluating healthcare interventions. Key to the success of any RCT is successful recruitment of participants. Less than one-third of publically funded trials manage to recruit their original target within the time originally specified and around one-third request additional funding and/or time [1]. Therefore, it is not surprising that more research papers (for example, [2-5]) are reporting on the many challenges of managing recruitment to RCTs and strategies to overcome them.

The POPPY (Pelvic Organ Prolapse PhysiotherapY) Trial was a multi-centre RCT which assessed the effectiveness and cost-effectiveness of individualised pelvic floor muscle training (PFMT) compared to a lifestyle advice leaflet for women with symptomatic pelvic organ prolapse. The main findings of this trial are published elsewhere [6]. This article describes the methodological challenges of recruiting to a multi-centre RCT of a complex intervention in a gynaecology setting, and the methods we used in an attempt to address them. These experiences and solutions may prove helpful for other large trials.

## Methods

The aim of the trial was to recruit 520 new gynaecology outpatients with symptomatic prolapse over a 16-month period from 14 UK hospitals and one New Zealand hospital. All the trial processes and documentation were approved by the relevant Ethics and Research and Development Committees and women gave signed informed consent to being randomised and to participate in long-term follow-up. Women were randomised by trial office staff using a central internet-based randomisation system to receive either PFMT (delivered by a physiotherapist who assessed and treated them at five appointments over 16 weeks and who prescribed a daily exercise programme), or to receive a Lifestyle Advice Sheet by post. The trial was overseen by an independent Trial Steering Committee (TSC) and a separate, independent Data Monitoring and Ethics Committee (DMEC).

We based our expected recruitment rate on data from our feasibility study (a pilot trial in two centres, completed in August 2005) [7]. Based on this information, we predicted that in a typical recruiting centre, at least three women per month would agree to be randomised, which would give 36 randomised women per centre over a 12-month recruitment period. However, in the main trial we encountered recruitment problems due to fewer eligible women presenting to participating clinics than predicted and women’s preference to receive active treatment such as pelvic floor muscle training, vaginal pessary (a device inserted vaginally to support the prolapse) or surgery. As a result of this lower recruitment rate, we explored a number of avenues in an attempt to improve recruitment and reach our recruitment target.

## Results

### Recruitment of participants

Gynaecologists completed a trial entry form for all new outpatient referrals to gynaecology if the referral letter indicated that the woman had prolapse symptoms. Information from the completed entry form indicated whether a woman was eligible for inclusion or not. The gynaecologist, or another nominated member of staff, then discussed trial participation with eligible women and provided them with a Patient Information Leaflet.

On receipt of the completed trial entry form a member of the research team from the central trial office contacted willing and eligible participants by telephone to seek their informed consent to trial participation. Following verbal consent, women were sent a consent form and baseline questionnaire to complete at home. Women were allowed three weeks to return their completed consent form and baseline questionnaire before being given a reminder telephone call. In total there were 146 baseline reminder telephone calls made; 68 women were contacted, and 72% went on to be randomised. Of the 78 who were not successfully contacted by telephone, a reminder letter and second copy of the baseline paperwork was sent. Forty-six percent of these women then went on to be randomised. The gynaecologists assessed prolapse staging using the Pelvic Organ Prolapse-Quantification system (POP-Q) [8] before randomisation and at 6 months post-randomisation. Women completed postal questionnaires at 6 and 12 months after randomisation, and a 24-month follow-up postal questionnaire was sent to a smaller number of women who consented to longer term follow-up (Table 1).

**Table 1:** Recruitment success by centre

**Centre number**	**Total randomised**	**Recruitment period (months)**	**Geographic location**	**Local researcher (Y/N)**
^a^1	130	30	Scotland	Y
2	0	23	Northern Ireland	N
3	12	27	Scotland	Y
4	0	0	England	Y
5	37	28	Scotland	Y
6	3	20	Scotland	Y
7	20	29	Scotland	Y
8	30	23	Scotland	Y
9	33	13	England	Y
10	6	27	Scotland	N
11	6	27	Scotland	Y
^a^12	38	30	Scotland	Y
13	1	28	Scotland	N
14	5	23	England	N
15	28	29	Scotland	N
16	0	30	Scotland	N
^c^17	19	33	New Zealand	Y
18	0	30	Scotland	N
19	7	22	England	N
^b^20	8	9	England	Y
^b^21	2	12	England	Y
^b^22	6	11	England	Y
^b^23	14	14	England	N
^b^24	7	13	England	Y
^b^25	6	13	England	Y
^b^26	3	12	England	Y
^b^27	0	9	England	N
^b^28	3	9	England	Y
^b^29	2	13	Northern Ireland	N
^b,c^30	22	24	Sydney	N

^a^Pilot centres. Recruitment numbers do not include women from the pilot study. ^b^New centre recruited as a result of trial extension. ^c^These centres were funded and managed separately from the UK centres, and recruitment started earlier in the New Zealand centre. Recruitment never started in centres 2, 4, 16, 18 and 27.

### Facilitators to recruitment

#### ***Trial extension***

**Figure 1:**
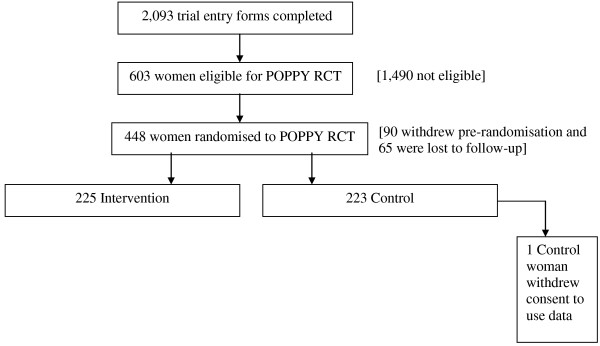
Overview of recruitment.

After 15 months of recruitment, when 175 women had been randomised, a request for additional funding and time was made to the funders to increase the duration of recruitment at existing centres by 12 months and to recruit additional centres. This extension was granted in February 2009 and allowed nine new UK centres and one extra international centre to come on board and recruit to the trial. Ultimately, 23 UK centres and two international centres (one in New Zealand and one in Australia, both of which were funded separately from the UK centres) recruited to the trial over a 30-month period. We finally randomised 448 women, of which one woman withdrew consent to using any of her data, leaving 447 (Figure 1).

#### ***Centre support***

The POPPY trial brought together a multidisciplinary group of hospital staff including gynaecologists, physiotherapists, nurses, secretaries and medical records. To ensure the trial was delivered according to the protocol and that accurate data were collected, it was important that everyone involved felt confident about the aims of the research and were adequately trained in the trial processes and procedures. Every UK centre had a site initiation visit from the trial team, bringing the relevant staff members together to fully describe the trial and the different roles and responsibilities of the local team members, to provide all trial materials and to confirm local trial arrangements. Subsequent visits were offered when necessary, and as a result, six centres had a second visit to further assist their participation in the trial. Many of the trial processes were embedded in existing clinical practice. For example, the POP-Q assessment method was already used by some gynaecologists when recording prolapse type and severity, and PFMT was an intervention routinely delivered by the majority of physiotherapists involved for women with urinary incontinence. Therefore, some components of the trial were familiar to the clinical collaborators, an important contributory factor in a successful trial [1].

#### ***Minimising centre workload***

Inevitably, RCTs generate extra work, both clinical and administrative, for centres involved. In order to minimise this burden, where possible we employed a dedicated local researcher (for example, a local nurse, physiotherapist or secretary with dedicated trial time) at participating centres to support recruitment and trial delivery more generally. The local researcher was able to facilitate the recruitment process by screening notes prior to clinics and attaching a data collection form to those of potentially eligible women for completion by the gynaecologist during clinic appointments. Having a dedicated person to aid recruitment in this way was helpful for both the recruiting gynaecologist and the trial office. For the recruiting gynaecologist it meant potentially eligible women were already highlighted to them indicating possible participation in the trial should be discussed with the woman, saving them time during busy clinics. For the trial office, having a local researcher meant there was access to a person at the centre who had dedicated time to assist with recruitment and who could be contacted directly to discuss problems.

We also, wherever possible, adapted the trial processes to fit in with existing local practice. This included intercepting clinical notes, as described above, having Patient Information Leaflets readily available in all recruiting clinics and displaying recruitment posters in both clinic and waiting room areas.

#### ***Involvement of general practitioners***

Following discussions with recruiting gynaecologists and local researchers, it became evident that many women were being referred from their general practitioner (GP) with a clear idea of what treatment they wanted to receive for their prolapse symptoms (for example, insertion of a vaginal pessary or prolapse repair surgery), making them less likely to consider participation in the trial. It was hypothesised that if women were counselled by their GP, who is generally their first point of contact, that the POPPY trial was another treatment option available at their hospital, then recruitment might improve. Thus involvement was sought from GP practices in areas feeding into trial centres.

In conjunction with the Scottish [9] and English [10] Primary Care Research Networks (PCRNs), we aimed to inform appropriate GPs (those who would normally refer to participating trial centres) of the trial. For example, if a GP suspected prolapse and the decision was to refer for a gynaecology outpatient appointment, the GP could address the referral letter to a recruiting gynaecologist. The GP would then inform the woman that the trial was available, and if she was eligible, the gynaecologist would discuss participation with her.

In order to do this, the trial had to be adopted by the Scottish and English PCRNs, and approvals from both Ethical and Research and Development for each participating centre were required. In reality, this was a very time-consuming process and was introduced too late to be of benefit. The trial started in April 2007 and stopped at the end of March 2011, including the 12-month extension. In Scotland, initial contact was made with the SPCRN in September 2008, 12 months after recruitment started, and agreement to the involvement of GPs was granted in April 2009. The approvals and set-up processes then took another four months, with GPs being sent information about the trial in August 2009, seven months before the end of recruitment.

In England, the process was more complex. Initial adoption by the network was relatively easy and quick, with our application for adoption being approved in June 2009, one month after submission. However, in addition to securing local Research and Development approval appropriate Primary Care Trust Research Management and Governance (PCT RM&G) approval had to be gained separately for each relevant geographical area. Applications for RM&G approvals were initiated in August 2009. In October 2009 we were informed we needed to secure service support costs to compensate GPs for their time to review the GP information leaflet and discuss the trial with prospective participants. It had been previously indicated in July 2009 that as the GP role was minimal it did not incur service support costs. This decision consequently delayed the approvals process whilst support costs were calculated and applications for funding were made. A decision was taken by the research team in November 2009 not to pursue further applications for PCT RM&G approvals and focus on the three centres for which approvals and support costs were in place.

### Barriers to recruitment

Recruitment proved to be more difficult than anticipated. In an attempt to identify factors affecting centre recruitment, the trial office engaged in regular discussions with local teams and a number of issues were highlighted.

#### ***Fewer eligible women than anticipated***

Many centres reported that fewer eligible women were coming through their clinics than originally anticipated. As part of the process of recruiting centres to the trial, data on patient throughput was requested in order to assess if sufficient women could be recruited to make the centre viable. Lasagna’s Law suggests the incidence of the disease studied in trials falls to 10% of the original estimate when recruitment begins due to over-estimations before recruitment starts [11]. Discussions with recruiting gynaecologists and local researchers suggested this may have been the case in some of the POPPY trial centres. For example, a centre which had over 3,000 new gynaecology outpatients the year prior to the grant application being submitted and expected to randomise three women per month actually randomised three women in total over a 20-month recruitment period. The ability to set up more centres to compensate for those which under-recruited was therefore vital.

#### ***Patient preference for pelvic floor muscle training***

Despite the lack of evidence for its effectiveness, some physiotherapists are already offering individualised PFMT for women who have small prolapses or are not good candidates for surgery [12]. Because the trial intervention was available outside the trial, some women withdrew once they discovered they had been allocated to the control group. Whilst it could be argued that these women should not have consented to participate as they were not in equipoise, this was difficult to ascertain at the time of recruitment and highlights the importance of exploring patient treatment preferences at the point of recruitment. Work carried out by Mills *et al*. (2011) [13] found discussion of treatment preferences at the point of recruitment did not act as a barrier to recruitment as expected, but allowed participants to highlight their concerns and make an informed decision that often included participation in the RCT.

#### ***Lack of support staff***

In centres that did not have a local researcher, screening was carried out by the recruiting gynaecologist, thus increasing the burden on a busy clinician. This may have had an impact on their ability to recruit.

#### ***Variations in approval systems***

The process of securing PCT RM&G turned out to be a lengthy process, with each RM&G department requiring different information.

Variations between Scottish and English GP referral procedures also affected the ability to involve GPs. At the time of recruiting, in Scotland a new software system called the SCI Gateway system [14] was introduced. This is a national system that integrates primary and secondary systems enabling GPs to make referrals electronically. At the time, individual health boards were using the system differently in that some wanted referrals sent electronically to a generic location which would then be dealt with by the health board, whilst others had individual clinicians named on the system so referrals could be made directly. This posed a major problem for recruitment. If a recruiting gynaecologist was not named on the system, GPs could not refer to him or her directly.

In England, the Choose and Book system was in place [15], a service that allows patients to choose which hospital or clinic they wish to attend following a GP appointment. As a result, the ability to refer potential participants to specific recruiting gynaecologists was outside the GP’s control.

#### ***Staff turnover***

Potential participants were recruited from gynaecology outpatient clinics. High staff turnover at these clinics due to staff rotation of gynaecology registrars had an impact on recruitment. The rotation resulted in continual training of new registrars of the trial protocol and processes, which impacted on our already limited time to recruit.

### Collaborator incentives

Sustaining motivation of collaborating centres to continue recruiting and of participants to stay committed to the trial can be a problem, particularly when the period of involvement is lengthy. Few studies report on incentives aimed at those individuals recruiting the trial participants [2]. To compensate the healthcare professionals for their time we paid a nominal amount of £55 for every woman randomised to the trial. These funds could be accessed by everyone involved in the trial at the recruiting centre to support future professional development such as attending courses and conferences. Whilst collaborating clinicians appeared enthused to receive this payment it did not appear to have a major impact on the level of recruitment, echoing the work carried out by Byrant and Powell [16].

**Figure 2:**
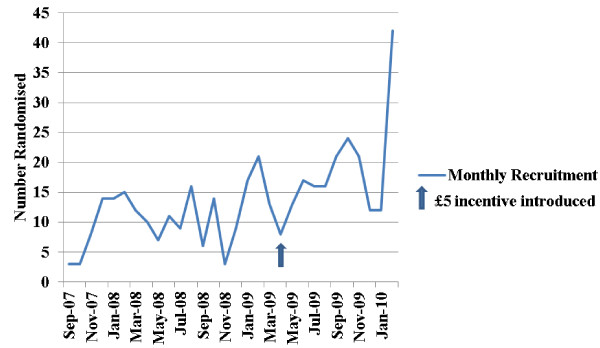
Overall monthly recruitment figures.

Individualised monthly recruitment targets were introduced for each recruiting centre in April 2009, 11 months before recruitment stopped. Importantly, these targets were realistic as they were based on previous recruitment rates for the particular centre, and thus reflected the local demographics. If centres met their targets, in addition to receiving the £55 per woman randomised, they were sent a £5 voucher to purchase refreshments such as coffee and biscuits for the local team. Interestingly, this approach appeared to have a more positive impact on recruitment at some centres than the £55 per woman randomised. Whilst only five centres met their recruitment targets every month, recruitment overall improved, and centres that had not recruited any women began to do so. Local researchers reported that the recruiting staff appeared to be more enthused by this incentive. A possible reason for this could be the staff most heavily involved in the research received the benefits of this incentive immediately, supporting Fletcher *et al.*’s [5] finding that all staff should be rewarded for participation in research, not just medical staff. Also, in some centres, the £55 per woman randomised did not reach the local team members as it was absorbed by the Research and Development departments. All research team members at each recruiting centre also received a trial mug and pen with the POPPY logo to heighten awareness of the trial (Figure 2).

### Communication

Overcoming trial fatigue experienced by both collaborators and participants requires endurance and commitment from everyone involved in a trial. Maintaining contact with centre staff and participants played an important role in the success of the POPPY trial. As POPPY was a national and international multi-centre trial, the main forms of contact were by e-mail and telephone. Regular feedback and updates from the trial office were sent to recruiting staff and participants to ensure they felt involved. These took the form of monthly email alerts to all centre staff detailing individualised recruitment updates and continually highlighting the importance of the trial, encouraging them to continue recruitment and acknowledging the investment of time and support they had contributed to date. They were also used to address any issues that arose regarding the trial processes and to provide updates on any protocol amendments.

## Discussion

Recruitment difficulties were encountered early on in the POPPY trial and additional funds and time were needed to extend both the recruitment period and the number of recruiting centres. Even with these additional resources, the target sample size of 520 was not achieved. We employed a number of strategies to try and improve recruitment and retention to the trial, and were guided in choice of strategies by the experience of the Project Management Group and Clinical Trial Unit, and the advice of the Trial Steering Committee, which had both clinician and consumer representation. Some were more successful than others, and they also differed in the resources required.

It is important for trialists to recognise that over-estimation of the number of potential participants available to take part in a trial is common. This must be taken into account when deciding how many centres will be required to deliver the chosen sample size. The impact of delays in centre start-up on recruitment should also be taken into account.

Minimising the impact of the trial processes on staff time at collaborating centres is important. We found centres with a dedicated local researcher who assisted both in recruitment and interacting with the trial office, recruited more successfully. Good communication within the whole research team (trial management team, collaborating centre staff and participants) and expression of appreciation at every opportunity is vital. As their participation is entirely voluntary, recognition and gratitude to participants as well as centre staff are essential.

Changing the recruitment process once a trial is under way results in the need for additional Ethical and Research and Development approvals. Even with standardised systems in place to streamline some of these processes, delays still occur, which affect the success of implementing the new strategies within the constrained timescale. It is therefore necessary to try and use strategies that are likely to be successful and which do not make excessive use of scarce resources for their implementation, hence the importance for trialists to report on their experiences of clinical trial recruitment. A notable example in this trial was the decision to encourage recruitment by involving GP practices from across health boards and health authorities in the different countries, which led to significant work for the trial office for little return. To our knowledge, no women were referred to a recruiting gynaecologist in Scotland or England following consultation with their GP for consideration of the POPPY trial. This approach may have been more successful if introduced at the start of the trial allowing more time for approvals to be obtained and more GPs to be informed about the trial.

We employed various centre staff and participant incentives, some of which appeared to be more successful than others. In our trial, friendly communication with participants by telephone appeared to have a positive impact on the return of baseline paperwork, attendance rates for 6-month prolapse assessments and the return of 6-month questionnaires. However, women’s involvement in the trial was still in the early stages at this point and they may have been eager to attend their 6-month review in any case, to see if there had been any change in their prolapse and seek further treatment, if appropriate. For recruiting centres, incentives whereby the staff benefited immediately, such as the £5 voucher to purchase refreshments, appeared to have a better impact on staff morale and recruitment rates than a contribution to departmental funds to be accessed at a later date for professional development needs. Trialists should therefore ensure a budget for small, appropriate incentives is included in grant applications, whenever possible.

In this narrative paper we have reported on our experiences of recruiting participants from a gynaecology setting and the different methods used in an attempt to improve recruitment and trial implementation. We acknowledge the limitations of narrative reflective articles and the importance of incorporating more systematic approaches to examining and addressing recruitment difficulties (for example, [2]), including the use of qualitative research [17] and the involvement of patient and public groups where possible. Using these methods in the feasibility stage could help inform the recruitment process of the main trial resulting in a more efficient trial. Whilst we did not test the methods discussed by formal RCT, some were clearly too time consuming or unproductive to be useful whilst others appeared promising such as incentives for staff.

## Conclusions

Successful recruitment of participants to a trial is central to its success and is dependants on the support and commitment of the whole research team. Poor recruitment can result in an underpowered study which may result in clinically important effects being missed. In an ideal world more time and resources would be assigned to the recruitment of collaborating centres and participants. However, constraints of most publicly funded research mean the amount of time to recruit is very limited. It is therefore important to have a recruitment and retention plan in place at the start of the trial to maximise recruitment.

Our article reflects on the methodological challenges of recruiting to a multi-centre RCT in UK gynaecology outpatient settings and the systems put in place in an attempt to address them. Trialists have a responsibility to publish their experiences of recruitment and lessons learned to improve the evidence base on clinical trial recruitment in different clinical areas and guide future trials. In addition, there is a need for future RCTs to evaluate, where possible, the effect of strategies designed to improve recruitment and retention.

## Abbreviations

DMEC: Data monitoring and ethics committee; GP: General practitioner; PCRNs: Primary care research networks; PCTRM&G: Primary care trust research management and governance; PFMT: Pelvic floor muscle training; POPPY: Pelvic organ prolapse physiotherapy trial; POP-Q: Pelvic organ prolapse-quantification system; RCTs: Randomised controlled trials; TSC: Trial steering committee.

## Competing interests

The trial was funded by the Chief Scientist Office (CSO) of the Scottish Government Health Directorates (project number CZH/4/377) and is summarised at http://www.cso.scot.nhs.uk/about/publications/focus-on-research-summaries/. NMAHP RU and HSRU are also funded by the CSO. However, the views expressed in this publication are those of the authors and not necessarily those of the CSO. The authors accept full responsibility for the research.

## Authors’ contributions

SD was the UK trial coordinator, was responsible for the day-to-day management of all aspects of the trial and the trial office, was responsible for centre initiation visits, and was responsible for the writing of the final manuscript. JL assisted with day-to-day UK trial management, liaised with centre staff, undertook reporting and contributed to the final manuscript. SH was the chief investigator of the study; had complete involvement in and oversight of the study design, execution, and data collection; and contributed to the final manuscript. DS contributed to the design of the trial overall and to the physiotherapy intervention specifically, was responsible for training the physiotherapists delivering the trial intervention, was responsible for centre initiation visits, and contributed to the final manuscript. CG contributed to the design of the study, its delivery, the choice and design of the outcomes measures, and contributed to the final manuscript. AMcD gave guidance on trial management throughout and contributed to the final manuscript. GMcP designed the programming of the study database and randomisation system and was involved in the data reporting. All authors read and approved the final manuscript.
